# Supplementary material for: Complexome profiling of the Chlamydomonas psb28 mutant reveals TEF5 as an early PSII assembly factor
Source: Plant Cell. 2025 Mar 17;37(6):koaf055. doi: 10.1093/plcell/koaf055 (PMC12164590; doi:10.1093/plcell/koaf055)
Supplement: koaf055_Supplementary_Data [file koaf055_supplementary_data.zip › psb28_interactive.zip!/plots/intensity/qualityPlots/peptides/TOM40_Cre10.g424450.t1.2.html]

### Cre10.g424450.t1.2

Trivial name: TOM40  
SeqCoverage [median]: 46.30  
Mass: 37.16 kDa  
  
 

| Sequence | MissedCleavage | Mass [Da] | wtIntensity | muIntensity | Score |
| --- | --- | --- | --- | --- | --- |
| AGPGGDSSPPPMPNFSFASQAAAVAK | 0 | 2458.16 | 4837.76 | 7480.93 | 116.65 |
| EGIISLKPDTFEGLR | 1 | 1673.90 | 3569.14 | 11293.68 | 71.88 |
| ESERPNYLELPQPVR | 1 | 1825.93 | 0.00 | 707.94 | 39.51 |
| ESERPNYLELPQPVRYEDIQR | 2 | 2630.31 | 1594.12 | 5176.25 | 59.28 |
| GGDWNAQLK | 0 | 987.48 | 283.39 | 0.00 | 64.27 |
| LGAPGFVGLNYFQSLTPK | 0 | 1908.01 | 2030.11 | 0.00 | 122.46 |
| LSAGGEFFYLPSNPK | 0 | 1625.81 | 4046.50 | 0.00 | 109.16 |
| SGVGLALR | 0 | 771.46 | 11266.61 | 18375.42 | 140.98 |
| TPVGTYEFGATTVSESQNLVLMGR | 0 | 2556.25 | 1099.21 | 0.00 | 67.56 |
| VQAQLANAANQSQVMLDTDVK | 0 | 2243.12 | 0.00 | 818.07 | 56.33 |
| YEDIQR | 0 | 822.39 | 287.95 | 1354.61 | 91.58 |
|  |  |  | 29014.8 | 45206.9 |  |
